# RapidPlan Knowledge-Based Radiotherapy Planning Compared to Manual Planning in Locally Advanced Non-Small-Cell Lung Cancer

**DOI:** 10.3390/cancers17223654

**Published:** 2025-11-14

**Authors:** Tal Falick Michaeli, Tamar Abu Said, Stanislav Raskin, Antoni Skripai, Yakir Rottenberg, Johnathan Arnon, Philip Blumenfeld

**Affiliations:** 1 Department of Radiation Oncology, Sharett Institute of Oncology Hadassah Medical Center, Jerusalem 91120, Israel; tamar.abusaeed@gmail.com (T.A.S.); stan@hadassah.org.il (S.R.); antoni.skripai@mail.huji.ac.il (A.S.); 2 Department of Developmental Biology and Cancer Research, Institute for Medical Research Israel-Canada, Hebrew University Medical School, Jerusalem 91120, Israel; 3 Department of Medical Oncology, Sharett Institute of Oncology Hadassah Medical Center, Jerusalem 91120, Israel; ryakir@hadassah.org.il (Y.R.); arnony@hadassah.org.il (J.A.)

**Keywords:** stage III NSCLC, RapidPlan (RP), organs-at-risk (OARs), IMRT, VMAT, dosimetric outcomes

## Abstract

**Background/Objectives:** Treatment planning for stage III non–small cell lung cancer (NSCLC) presents dosimetric challenges due to the proximity of critical structures. RapidPlan (RP), a knowledge-based planning (KBP) system, offers the potential for improved plan consistency and organ-at-risk (OAR) sparing. The objective of this study was to compare dosimetric and clinical outcomes of RP-generated plans versus manually optimized plans in patients with stage III NSCLC undergoing IMRT or VMAT. **Methods:** In this retrospective analysis, 50 patients treated with concurrent chemoradiation for stage III NSCLC at Hadassah Medical Center (2015–2021) were analyzed. RP plans were generated using a lung-specific model in the Eclipse treatment planning system and compared with the original clinical manual plans. Dosimetric parameters for target volumes and OARs were evaluated, and subgroup analyses were performed by technique (IMRT vs. VMAT). Toxicity and survival outcomes were analyzed, and Normal Tissue Complication Probability (NTCP) modeling was conducted. **Results:** RP significantly reduced mean heart dose (Δ = −2.54 Gy, *p* < 0.001), spinal cord maximum dose (Δ = −4.08 Gy, *p* < 0.001), and esophageal mean dose (Δ = −3.89 Gy, *p* < 0.001) compared with manual plans. Lung doses were slightly higher in RP plans (V20 Δ = +2.12%, *p* < 0.001). VMAT-RP plans demonstrated greater cardiac and esophageal sparing than VMAT-manual plans. RP yielded significant NTCP reductions for the heart (0.34% → 0.20%) and esophagus (16.6% → 11.5%), but no improvement for lung or spinal cord. Lung toxicity ≥ grade 2 was associated with reduced overall survival (16.2 vs. 51.8 months, *p* < 0.001). **Conclusions:** RapidPlan-based knowledge-based planning enhances OAR sparing while maintaining target coverage in locally advanced NSCLC. Slight increases in lung dose highlight the need for ongoing model refinement. An association between lung toxicity and reduced survival was observed, underscoring the impact of treatment-related morbidity on outcomes.

## Simple Summary

We retrospectively analyzed 50 patients with stage III NSCLC treated with concurrent chemoradiation between 2015 and 2021 to compare knowledge-based planning (RP) with manually optimized IMRT/VMAT plans. RP achieved comparable target coverage while significantly reducing mean doses to the heart (−2.5 Gy), esophagus (−3.9 Gy), and spinal cord (−4.1 Gy), though with a modest increase in lung V20 (+2.1%). Normal tissue complication probability estimates predicted lower cardiac and esophageal toxicity with RP but no clear improvement for lung or spinal cord. Clinically, lung toxicity ≥ grade 2 strongly correlated with inferior overall survival (16.2 vs. 51.8 months, *p* < 0.001). RP thus enhanced organ-at-risk sparing, particularly for cardiac and esophageal structures, but required attention to pulmonary dose trade-offs. These findings underscore both the potential and the limitations of knowledge-based planning and highlight the need for individualized optimization strategies in stage III NSCLC radiotherapy.

## 1. Introduction

Lung cancer remains the leading cause of cancer-related mortality in developed countries, with non-small-cell lung cancer (NSCLC) accounting for approximately 85% of cases [1]. Stage III disease, affecting nearly one-quarter of NSCLC patients, represents a significant therapeutic challenge [1]. According to the World Health Organization (WHO), NSCLC is broadly classified into adenocarcinoma (≈40% of cases), squamous cell carcinoma, and large cell carcinoma, each with distinct clinical and pathological features [2]. The eighth edition of the TNM staging system further divides stage III into IIIA, IIIB, and IIIC, based on tumor size, invasion of adjacent structures, and nodal involvement [3]. While stage III NSCLC may be curable, unresectable cases are associated with poor outcomes, with a 5-year overall survival (OS) ranging from 19% to 36% [1].

The standard of care for unresectable stage III NSCLC has been concurrent chemoradiotherapy (CRT) with platinum-based agents, typically delivering 60–66 Gy in 2 Gy fractions [1]. Despite local control rates of up to 70%, progression-free survival (PFS) and OS remain suboptimal [4]. A paradigm shift occurred with the PACIFIC trial, which demonstrated that adjuvant durvalumab after CRT significantly improved outcomes, yielding an 11.2-month gain in median PFS and 15–20% higher PFS rates at 12 and 18 months [5]. Locoregional recurrence continues to pose a significant challenge, affecting over 30% of patients [6]. Attempts at dose escalation have proven counterproductive, as evidenced by the RTOG 0617 trial, where escalation to 74 Gy yielded inferior outcomes compared to the standard 60 Gy regimen [6]. Furthermore, cardiac radiation exposure has emerged as a crucial independent prognostic factor in stage III NSCLC [7]. Research has revealed a direct correlation between cardiac radiation dose and radiation-induced cardiac toxicity, subsequently impacting overall survival. These findings underscore the importance of treatment planning techniques that optimize cardiac sparing while maintaining adequate tumor coverage [7,8].

RapidPlan (RP), a sophisticated knowledge-based planning (KBP) system, represents an innovative approach to enhancing radiotherapy planning quality, consistency, and efficiency through its utilization of a high-quality case database [9]. The system generates predicted dose–volume histograms (DVHs) for organs at risk (OARs) and provides optimization objectives to guide the planning process [9,10]. By integrating geometric and dosimetric parameters, RP offers enhanced organ protection, reduced planning time, and improved plan quality. Its effectiveness has been validated across various malignancies, including prostate cancer and mesothelioma [11,12,13]. Recent work has shown that the quality of the training dataset significantly impacts model performance. For example, Fjellanger et al. demonstrated that RapidPlan models trained on multi-criteria optimized (MCO) plans produced superior dosimetry compared with models trained on standard clinical plans in LA-NSCLC [13]. Similarly, Seng et al. reported that laterality-specific RapidPlan models (right vs. left lung) yielded measurable improvements over general lung models, highlighting the potential benefit of tailoring KBP models to anatomical subgroups [14]. Comparative studies of vendor-provided automation have also been performed. Shao et al. described the development of an in-house KBP system that automatically generated IMRT plans for lung cancer, demonstrating both robust plan quality and substantial workflow efficiency gains [15]. Finally, Harms et al. highlighted practical aspects of rapid implementation and automated evaluation of RapidPlan models, providing a framework for clinical commissioning and QA [16]. Collectively, these studies underscore the potential of KBP to enhance plan quality, reduce variability, and streamline clinical workflows in NSCLC radiotherapy. This study seeks to evaluate the comparative dosimetric outcomes between RP-based and manual planning methodologies in the context of volumetric modulated arc therapy (VMAT) and Intensity-Modulated Radiation Therapy (IMRT) for locally advanced NSCLC, with particular emphasis on assessing improvements in target coverage and OAR sparing that could potentially enhance clinical outcomes for stage III NSCLC patients. Toxicity and survival outcomes were also assessed to investigate any correlation between dosimetric and patient outcomes.

## 2. Methods

This retrospective observational study analyzed 50 patients with stage NSCLC (T1-T4, N1-N3, M0) treated at the Sharett Institute of Oncology, Hadassah Medical Center between 1 January 2015, and 1 January 2021.

Inclusion criteria comprised patients with histologically confirmed non-small-cell lung cancer (NSCLC) stages T1–T4, N1–N3, M0, treated with definitive concurrent chemoradiation therapy (60–70 Gy in 2 Gy fractions) using IMRT or VMAT techniques between 1 January 2015, and 1 January 2021, at the Sharett Institute of Oncology, Hadassah Medical Center. Patients were required to have complete clinical, imaging, and dosimetric data available in the institutional electronic medical record. Exclusion criteria included patients with distant metastases (M1), prior thoracic irradiation or re-irradiation, non-definitive or palliative treatment intent, incomplete radiation courses, non-standard techniques (e.g., 3D-CRT, SBRT, or proton therapy), recurrent disease, missing or inadequate planning data, or significant pre-existing cardiopulmonary comorbidities that could confound toxicity assessment.

For comparative analysis, new treatment plans were generated using a commercially available knowledge-based feedback RP model developed by ORBIT-RT (On-line Real-time Benchmarking Informatics Technology for RadioTherapy) specifically designed for locally advanced lung cancer. RP models are constructed by reviewing retrospective clinically treated cases based on commonalities in clinical variables such as modality, treatment sites, prescription dose, and extent of disease. The plans quality, including organs-at-risk (OAR) sparing, target coverage, conformality, and homogeneity, were assessed by dosimetrists based on clinical experience. Suboptimal cases and those considered atypical (e.g., unique setups and target location, recurrent treatment) were filtered out. The RP model for the Lung/Mediastinum was constructed using 78 external cases and validated using 25 external cases.

Dosimetric parameters were evaluated between the original manual plans and RP-generated plans, including target coverage metrics for Gross Tumor Volume (GTV), Clinical Target Volume (CTV), and Planning Target Volume (PTV). PTV margins were standardized across all patients and across all plans (Manual and RP) and set to be 0.5 cm from the CTV based on local standards used in the clinic. Organ-at-risk doses were assessed for heart (mean dose, V50, V40, V25), lung (V20, V5), spinal canal (maximum dose), and esophagus (mean and maximum dose).

All treatment plans were generated using the Eclipse treatment planning system (TPS). Both RP and manually optimized plans were planned with the VMAT technique. Manual plans were performed with varying arc setups across all patients (partial arcs, half arcs, full arcs); each arc setup was chosen to be the most suitable for a given patient by considering anatomy structure, tumor size, and tumor location. RP-based planning was performed using two half arcs (0–180°) for most patients. However, in select cases where GTV location and size resulted in suboptimal dosimetric outcomes, two full arcs were used instead. A total of 21 patients received a replan after 44 Gy in addition to their primary plan. In such cases, separate RP plans were created for the primary and replan treatments. Dose evaluation was conducted by analyzing the combined dose distribution of both plans simultaneously using the “plan sum” feature in Eclipse TPS.

The study also assessed multiple clinical outcomes, including disease control parameters (locoregional control within radiation field, regional control outside radiation field, distant control for metastases), treatment-related toxicities (cardiac complications including pericardial effusion, ischemia, arrhythmias, and heart failure; pulmonary toxicity including pneumonitis and lung fibrosis), and survival metrics (PFS and OS).

To assess the clinical relevance of dosimetric differences, normal tissue complication probability (NTCP) estimates were calculated using the Lyman–Kutcher–Burman (LKB) model. NTCP was modeled for three organs at risk: lung (radiation pneumonitis), heart (cardiac toxicity), and esophagus (radiation-induced esophagitis), based on QUANTEC-recommended parameters.

The following model parameters were used:

- Lung: TD_50_ = 30.8 Gy, m = 0.45
- Heart: TD_50_ = 52.0 Gy, m = 0.28
- Esophagus: TD_50_ = 47.0 Gy, m = 0.36

Mean doses were used for the lung, heart, and esophagus NTCP calculations. The spinal cord was evaluated dosimetrically using maximum point dose, but NTCP modeling was not included due to low exposure levels.

Statistical analysis included comparison of dosimetric parameters, with significant differences defined as relative differences ≥10% between clinical and RP plans (with 95% confidence intervals). We have selected a 10% threshold to represent a clinically meaningful dosimetric change as consistent with established practice in knowledge-based planning literature, where differences below this magnitude are generally considered to fall within the range of inter-planner variability and are less likely to be clinically significant.

Survival analysis utilized Kaplan–Meier analysis with log-rank test for categorical variables and Cox regression for continuous variables including age, GTV, and dosimetric parameters. Quantitative variables were presented as mean ± standard deviation (SD) and median with ranges, while categorical variables were expressed as frequencies and percentages. Non-parametric analysis employed Mann–Whitney test and Wilcoxon signed-rank test, while categorical associations were evaluated using Fisher’s exact test. We used IBM SPSS Statistics, 22nd version for statistical analysis.

This study was conducted in accordance with the Declaration of Helsinki and received appropriate institutional ethical approval (IRB number HMO-0736-21). The primary objectives were to compare dosimetric outcomes between manual and RP-generated IMRT/VMAT plans, evaluate the potential of RP to maintain target coverage while reducing OAR doses, assess planning efficiency, and investigate correlations between cardiopulmonary toxicities and OS.

## 3. Results

### 3.1. Patient Demographics and Disease Characteristics

As shown in Table 1, among the 50 patients included in this study, stage IIIA disease was predominant (64%), followed by IIIB (24%) and IIIC (12%). The majority of tumors were located in the right upper lobe (44%), with remaining cases distributed across the right middle lobe (10%), right lower lobe (16%), left upper lobe (18%), and left lower lobe (14%). Adenocarcinoma was the predominant histology (58%), followed by squamous cell carcinoma (40%), with histology unavailable for one patient (2%). The majority of evaluable patients (56%) lacked EGFR or ALK mutations. Other molecular alterations were identified in 12% of patients. Most patients demonstrated good performance status, with 66% being ECOG 0 and 28% ECOG 1. All patients had a current or past smoking history. Regarding comorbidities, cardiac conditions were most prevalent (36%), followed by hypertension (20%) and hyperlipidemia (10%). Pre-existing lung conditions were noted in 13% of patients. Remaining characteristics can be viewed in Table 1.

### 3.2. Treatment Characteristics

All except for one patient received concurrent chemoradiotherapy. Mean radiotherapy dose was 60.96 Gy. In 22 patients, planned offline adaptive radiotherapy was performed after 44 Gy with a boost to 60–66 Gy to an adapted PTV. Carbo–taxol was the primary concurrent chemotherapy regimen (68%), while 6% were unknown or had no concurrent chemotherapy; Carbo–alimta (12%) and cisplatin–navelbine (10%) comprised the remaining regimens. Durvalumab was administered to 52% of patients following chemoradiotherapy. Table 1 and Table 2.

### 3.3. Dosimetric Analysis

As shown in Table 2 and Table 3, the dosimetric comparison between Manual Treatment Plans and RPs highlights key differences in OAR doses and target coverage. The Manual Plan demonstrated a slightly higher mean lung dose (16.25 Gy vs. 16.91 Gy in RP) and Lung V20 (27.52% vs. 29.66%). The Mean Heart Dose was lower in the RP (9.99 Gy vs. 12.54 Gy). Similarly, the spinal cord maximum dose (Cord Max) was lower in the RP (38.85 Gy vs. 42.92 Gy). Esophageal doses were also reduced in the RP, with lower mean (26.67 Gy vs. 30.56 Gy) and maximum dose (Dmax) (59.69 Gy vs. 62.01 Gy). Dmax and its percentage were slightly lower in the RP (66.23 Gy vs. 67.38 Gy, and 108.71% vs. 110.59%, respectively), indicating better dose conformity. Notably, the GTV minimum dose remained nearly identical in both approaches (~58.5 Gy), ensuring target coverage was not compromised.

### 3.4. Treatment Technique Subanalysis

Detailed analysis using the Wilcoxon Signed Ranks Test analysis of planning techniques revealed distinct patterns between VMAT and IMRT implementations can be seen in Table 4. Compared to manual VMAT plans, RP achieved statistically significant reductions in critical structure doses while maintaining similar lung parameters. Specifically, RP plans demonstrated mean heart dose reductions of 2.115 Gy (*p* < 0.01), spinal cord Dmax reductions of 4.751 Gy (*p* < 0.01), and esophageal mean dose reductions of 3.283 Gy (*p* < 0.01) compared to manual VMAT plans. Lung V20 and mean lung dose differences were not statistically significant in VMAT plans (*p* = 0.102 and *p* = 0.409, respectively). RP showed more pronounced differences when compared to manual planning with IMRT, RP achieved significant reductions in heart doses (mean reduction 3.1 Gy, *p* < 0.001) and spinal cord maximum doses (reduction 3.143 Gy, *p* < 0.01), resulting in higher lung doses. Lung V20 increased by 3.766 (*p* < 0.01) and mean lung dose increased by 1.282 Gy (*p* = 0.01) compared to manual IMRT plans. Esophageal sparing was also more pronounced when compared to IMRT plans, with mean dose reductions of 4.72 Gy (*p* < 0.01).

### 3.5. Toxicity and Survival Outcomes

**Figure 1:**
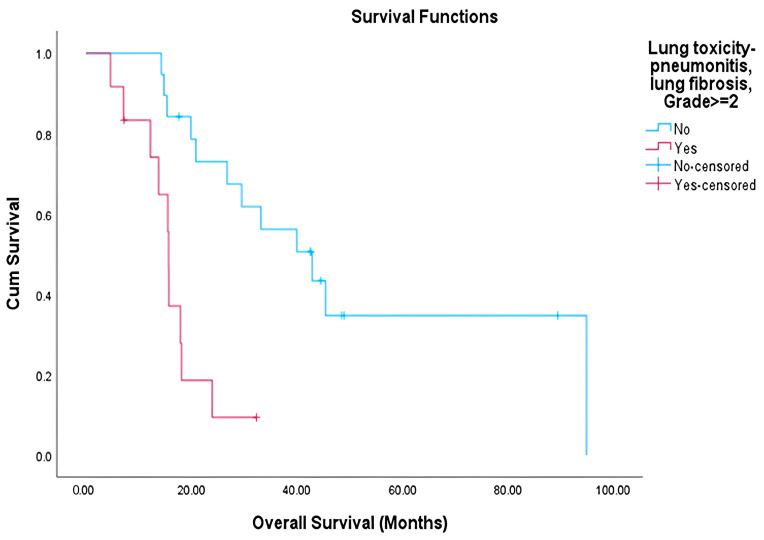
Overall survival by lung toxicity grades.

**Figure 2:**
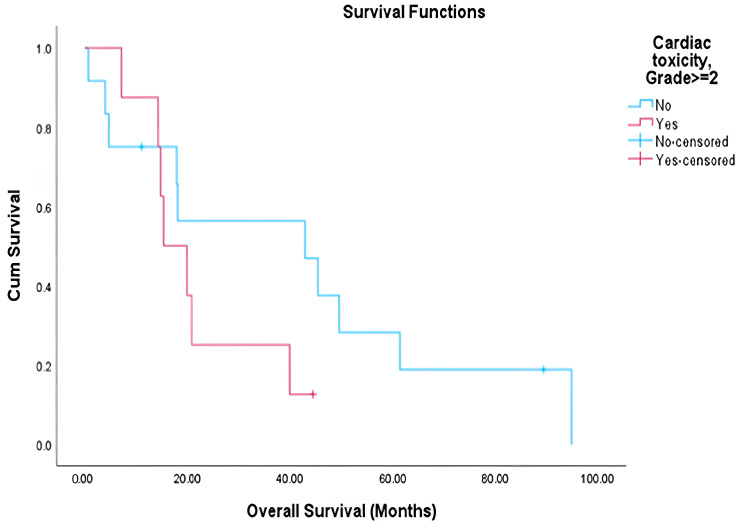
Overall survival by cardiac toxicity grades.

Among the evaluable patients, grade ≥ 2 pulmonary toxicity was observed in 38.7% (12 of 31 patients), while grade ≥ 2 cardiac toxicity occurred in 40% (8 of 20 patients) (Table 5). Patients who developed these toxicities had marginally higher mean heart doses compared to those without toxicity, though this difference was not statistically significant (Table 6). Survival analysis demonstrated a significantly shorter overall survival (OS) in patients who developed lung toxicity (mean OS: 16.22 vs. 51.75 months, *p* < 0.001) (Figure 1). A similar trend was noted among patients with cardiac toxicity (mean OS: 22.04 vs. 40.58 months), though this did not reach statistical significance (*p* = 0.207) (Figure 2). The median OS for the entire cohort was 23.89 months, with a mean OS of 39.84 months. No significant correlations were identified between the dosimetric parameters analyzed and either toxicity or survival outcomes.

### 3.6. NTCP Estimates

As shown in Table 7, NTCP modeling showed a slight, non-significant increase in lung toxicity with RP (15.8% vs. 14.7%, *p* = 0.33). In contrast, RP significantly reduced predicted heart toxicity (0.20% vs. 0.34%, *p* < 0.001) and esophageal toxicity (11.5% vs. 16.6%, *p* < 0.001). Spinal cord NTCP remained negligible in both plans.

## 4. Discussion

This study highlights the potential advantages of KBP using RP in the radiotherapy management of stage III NSCLC. Our findings demonstrate that RP maintains adequate target coverage while significantly reducing radiation exposure to key OARs. Specifically, RP resulted in a mean heart dose reduction of 2.54 Gy, a spinal cord maximum dose reduction of 4.07 Gy, and a mean esophageal dose reduction of 2.45 Gy. These results are consistent with prior studies, reinforcing the value of KBP in enhancing plan quality and consistency [17]. However, the absence of lung dose improvement, and the observation of slightly higher V20 values in some RP plans, raises important considerations for optimization strategies.

Our results align with growing evidence supporting RP efficacy. For example, Harms et al. demonstrated similar OAR dose reductions in a multi-institutional analysis of 31 NSCLC cases using RP, with meaningful reductions in mean dose to the heart and cardiac substructures (1.4 Gy to chambers, 3.3 Gy to coronary arteries, and 3.0 Gy to valves) [18]. Additionally, the quality of the model itself is a critical factor: models built from suboptimal or non-standardized clinical plans may yield suboptimal output, emphasizing the importance of carefully curated and optimized training data for KBP model development [19].

Our subgroup analysis also revealed a differential benefit between VMAT and IMRT. RP applied to VMAT demonstrated superior performance in heart sparing and overall OAR dose reduction, suggesting VMAT may be the preferred delivery technique in stage III NSCLC. These findings are consistent with prior reports, such as the report by Zhou et al., who showed that POA-VMAT enabled the creation of high-quality plans in cervicothoracic esophageal cancer without increasing OAR dose compared to manual planning [20].

Importantly, our results underscore the clinical relevance of radiation-induced toxicity. In RTOG 0617, grade ≥ 3 pneumonitis rates were similar between standard- and high-dose arms, but a secondary analysis showed IMRT significantly reduced severe pneumonitis compared with 3D-CRT (3.5% vs. 8.2%, *p* = 0.03), though overall survival was worse in the high-dose arm [20,21]. The PACIFIC trial reported that 3.4% of patients in the durvalumab arm and 2.6% in the placebo arm experienced grade 3–4 pneumonitis. In our cohort, we report lung toxicity of ≥grade 2 that was significantly associated with reduced overall survival (median 15.6 vs. 42.8 months, *p* < 0.001), supporting the growing consensus that treatment-related toxicity is a key determinant of long term outcomes [17]. While cardiac toxicity showed a similar trend, statistical significance was not achieved, possibly due to sample size limitations and follow-up constraints. Although we did not observe significant associations between specific dosimetric parameters and toxicity in our cohort, likely due to sample size, multiple previous studies have established such correlations [20,21].

A notable trade-off observed in RP is the inverse relationship between heart and lung dose. While RP reduces heart dose effectively, this is sometimes achieved at the expense of increased low-dose lung exposure (higher V5), raising concern given the known association of both V5 and V20 with pulmonary toxicity. In our experience, this shift raises questions about the optimal balance. One potential strategy could involve removing V5 from the optimization constraints, permitting a controlled increase in low-dose lung exposure while tightening restrictions on V20. This may facilitate a more deliberate and clinically relevant dose distribution, reducing moderate-dose lung exposure, which is more strongly associated with symptomatic pneumonitis. However, the true clinical benefit of this trade-off remains to be validated and may require personalized adjustments.

While our study did not evaluate individual cardiac substructures, other studies have explored this approach to further delineate cardiac toxicity and refine dose toxicity relationships. The recent literature has shown that mean heart dose alone may not fully capture radiation-related cardiac risk. Several studies have demonstrated that dose to specific cardiac components, such as the coronary arteries, right atrium, and aortic root, is more predictive of cardiac morbidity and survival [22,23,24]. Key challenges identified include the complexity of accurately delineating substructures and inter-operator variability. Automatic contouring tools now enable reproducible identification of these substructures and incorporating them into treatment planning has shown dosimetric and potential clinical benefits [23,24].

This retrospective, single-center study has inherent limitations, including potential selection bias, limited generalizability, and a modest cohort size. The restricted follow-up further limited assessment of toxicity outcomes, and the lack of real-time clinical integration of RapidPlan’s influence on treatment decisions constrains the applicability of our findings. Of note, NTCP estimates were derived using QUANTEC-based parameters for pneumonitis, esophagitis, and cardiac events, widely accepted reference models in thoracic radiotherapy, selected to ensure standardized comparison between RapidPlan and manually generated plans. However, as these models were developed from historical cohorts and KBP performance depends on training data quality, these factors represent additional limitations [25]. Looking forward, future efforts should aim to validate these findings in larger, prospective, multi-institutional studies. Incorporating artificial intelligence (AI) and radiomics into treatment planning holds promise for improving personalization. For example, a secondary analysis of the RTOG 0617 trial employed machine learning and explainable AI to identify dose–toxicity relationships [26]. That study found that mean lung dose > 18 Gy and V20 > 37% were strong predictors of grade ≥ 3 pulmonary toxicity, outperforming conventional models. The use of SHAP (Shapley additive explanations) enabled transparent interpretation of these predictions, further supporting their clinical relevance [27,28]. Similarly, radiomics analysis has shown potential in guiding dose adaptation, predicting patient-specific risk, and refining prescription strategies [29,30].

## 5. Conclusions

Knowledge-based planning with RP improved plan quality and reduced cardiac, esophageal, and spinal cord doses in stage III NSCLC, while maintaining target coverage. A modest increase in lung dose highlights the need to balance cardiac and pulmonary sparing. Lung toxicity ≥ grade 2 correlated with poorer survival, underscoring its clinical significance. RP offers a valuable planning tool, but individualized optimization and integration of AI and radiomics are needed to further refine dose–toxicity relationships and improve outcomes.

## Figures and Tables

**Table 1:** Patient characteristics and treatment details.

Characteristics	Value	Percentage (%)
Sex		
Male	42	84
Female	8	16
Performance Status ECOG		
0	33	66
1	14	28
2	3	6
Cardiac comorbidities	18	36
Lung comorbidities	6	12
Stage		
IIIA	32	64
IIIB	12	24
IIIC	6	12
T stage		
Stage1	3	6
Stage2	12	24
Stage3	23	46
Stage4	12	24
N stage		
N0	2	4
N1	4	8
N2	33	66
N3	11	22
Histology		
Squamous	20	40
Adenocarcinoma	29	58
Unknown	1	2
PDL1		
<1%	19	38
1–49%	4	8
>50%	5	10
Unknown/not performed	22	44
Type of concurrent chemotherapy		
Carbo–taxol	34	68
Carbo–alimta	6	12
CIS Navelbine	5	10
Carbo–pemetrexed	2	4
Unknown	2	4
None	1	2
Durvulumab		
Yes	26	52
No	16	32
**Unknown**	8	16

Abbreviations: ECOG = Eastern Cooperative Oncology Group. 0—Fully active. 1—Strenuous physical activity restricted. 2—Capable of all self-care but unable to carry out any work activities; EGFR = epidermal growth factor receptor; PDL1 = programmed death ligand 1.

**Table 2:** RP and MP variables.

Manual Treatment Plan Variables	Mean, [95% CI]	Range
PTV	700.75, [529.09, 809.41]	[103.4–1931.29]
Total Dose	69.96, [69.37, 70.55]	[58–66]
Lung V20	27.52, [25.47, 29.57]	[3–45.6]
Mean Lung	16.25, [15.28, 17.22]	[8.55–24]
Mean Heart dose	12.54, [10.25, 14.83]	[0.45–27.75]
Cord Max	42.92, [40.72, 45.12]	[23.7–56.58]
Esophagus Mean	30.56, [28.26, 32.86]	[16–50.2]
Esophagus Max	62.01, [60.85, 63.71]	[47.7–68]
Dmax	67.38, [66.64, 68.12]	[61.5–75]
Dmax %	110.59, [109.44, 111.74]	[101.72–125]
GTV Min dose	58.53, [57.09, 59.97]	[31.44–66.46]
GTV Min dose %	96.33, [93.97, 98.69]	[49.12–104.5]
Rapid Plan Variables	Mean, [95% CI]	Range
Lung V20	29.66, [27.23, 32.09]	[5–48]
Mean Lung	16.91, [15.80, 18.02]	[9.1–27]
Mean Heart dose	9.99, [8.35, 11.63]	[0.41–19.81]
Cord Max	38.85, [35.90, 41.80]	[16.83–61.13]
Esophagus Mean	26.67, [23.97, 29.37]	[11.5–61.39]
Esophagus Max	59.69, [57.78, 61.60]	[27.38–70]
Dmax	66.23, [65.18, 67.28]	[50.6–76]
Dmax %	108.71, [107.07, 110.35]	[76.67–119.21]
GTV Min dose	58.53, [57.57, 59.49]	[52–67.51]
GTV Min dose %	96.27, [94.88, 97.66]	[81.24–112.51]

Abbreviations: PTV = Planning Target Volume; Lung V20 = the percentage of normal lung receiving at least 20 Gy; Dmax = maximum point dose to an organ or tumor target in radiotherapy cancer treatment; GTV = Gross Tumor Volume.

**Table 3:** Dosimetric comparison (Delta of mean) between RP and MP variables.

[Parameter in RP—Parameter in MP]	Mean	95% CI	One-Sided *p*-Value
Lung v20	2.12	[0.990, 3.274]	0.000
Mean lung	0.66	[0.212, 1.114]	0.002
Mean heart dose	−2.54	[−3.478, −1.611]	0.000
Cord max	−4.08	[−6.184, −1.968]	0.000
Esophagus mean	−3.89	[−5.668, −2.106]	0.000
Esophagus max	−2.31	[−4.063, −0.563]	0.005
Dmax	−1.15	[−2.167, −0.128]	0.014
Dmax%	−1.88	[−3.500, −0.252]	0.012
GTV min dose	0.003	[−1.670, 1.663]	0.498
GTV min dose %	−0.06	[−3.400, 1.579]	0.482

Abbreviations: RP = Rapid plan; MP = manual plan; CI = Confidence Interval [upper limit, lower limit].

**Table 4:** Dosimetric comparison (Delta of mean) between RP and MP variables divided by VMAT and IMRT groups.

	VMAT (29 Patients)			IMRT (21 Patients)		
[Parameter in RP—Parameter in MP]	Mean	*p*-Value	95% CI	Mean	*p*-Value	95% CI
Lung v20	0.948	0.102	[−0.196, 2.094]	3.766	0.002	[1.629, 5.904]
Mean lung	0.214	0.409	[−0.175, 0.604]	1.282	0.010	[0.370, 2.195]
Mean heart dose	−2.115	0.000	[−3.053, −1.179]	−3.137	0.002	[−5.030,−1.244]
Cord max	−4.751	0.002	[−7.651, −1.852]	−3.143	0.068	[−6.422, 0.135]
Esophagus mean	−3.283	0.001	[−6.092, −0.476]	−4.720	0.000	[−6.688, −2.751]
Esophagus max	−3.534	0.002	[−6.368, −0.702]	−0.626	0.664	[−2.071, 0.819]
Dmax	−0.372	0.387	[−1.270, 0.525]	−2.218	0.027	[−4.337, −0.099]
Dmax%	−0.627	0.356	[−2.118, 0.863]	−3.601	0.027	[−6.919, −0.283]
GTV min dose	−0.534	0.295	[−2.030, 0.961]	0.852	0.381	[4.719, 0.465]
GTV min dose %	−0.910	0.284	[−3.400, 1.579]	1.308	0.381	[7.448, 0.449]

Abbreviations: RP = Rapid plan; MP = manual plan; VMAT = volumetric modulated arc therapy; IMRT = intensity-modulated radiation therapy; Wilcoxon Signed Ranks Test: used to compare two sets of scores that come from the same participants; CI = Confidence Interval [upper limit, lower limit].

**Table 5:** Lung and cardiac toxicity for patients with or Grade ≥ 2 in CTCAE v5.0.

	Grade ≥ 2	Percentage *	Mean ^a^ of OS (Months), [CI]	*p*-Value ^b^
Lung Toxicity				0.000
Yes	12	38.7	16.22, [12.078, 20.354]	
No	19	61.3	51.75, [35.101, 68.395]	
Total	31		39.84, [27.121, 52.570]	
Unknown	19			
Cardiac Toxicity				0.207
Yes	8	40	22.04, [13.504, 30.578]	
No	12	60	40.58, [20.578, 60.581]	
Total	20		33.81, [20.164, 47.449]	
Unknown	30			

Abbreviations: CTCAE = Common Terminology Criteria for Adverse Events; Lung toxicity = Lung toxicity—pneumonitis, lung fibrosis, etc. Grade ≥ 2; OS= overall survival; * percentage from valid N; a = estimation is limited to the largest survival time if it is censored. b = Log Rank (Mantel–Cox)-test of equality of survival distributions for the different levels of lung and cardiac toxicity, Grade ≥ 2; Unknown = patients who had no follow-up recorded; CI = Confidence Interval [upper limit, lower limit].

**Table 6:** Dosage for patients with Grade ≥ 2 in CTCAE v5.0 by cardiac toxicity.

Patients with Grade ≥ 2 Cardiac Toxicity Had a Slightly Higher Mean Heart Dose Compared to Those Without.				
	Grade ≥ 2	Mean Heart Dose	95% CI	*p*-Value
Cardiac Toxicity				0.7
Yes	8	13.12 Gy	[6.72, 19.52]	
No	12	12.02 Gy	[7.40, 16.64]	
Total	20			
unknown	30			

Mann–Whitney U test: a non-parametric test used to compare two independent groups.

**Table 7:** NTCP Analysis: Manual vs. RapidPlan.

Organ	Plan Type	Dose Type	Dose (Gy), [95% CI]	Estimated NTCP (%)	Δ NTCP (vs. Manual)	Statistical Significance (*p*-Value)
Lung	Manual	Mean	16.25, [15.28, 17.22]	14.7	-	Reference
Lung	RapidPlan	Mean	16.91, [15.80, 18.02]	15.8	+1.1	ns (*p* = 0.33)
Heart	Manual	Mean	12.54, [10.25, 14.83]	0.34	-	Reference
Heart	RapidPlan	Mean	9.99, [8.35, 11.63]	0.2	−0.14	*p* < 0.001
Esophagus	Manual	Mean	30.56, [28.26, 32.86]	16.6	-	Reference
Esophagus	RapidPlan	Mean	26.67, [23.97, 29.37]	11.5	−5.1	*p* < 0.001
Spinal Cord	Manual	Max	42.92, [40.72, 45.12]	0.0	-	NTCP ~ 0
Spinal Cord	RapidPlan	Max	38.85, [35.90, 41.80]	0.0	0.00	NTCP ~ 0

## Data Availability

The research data is securely stored by the research team and will be shared upon request to the corresponding author.
